# Supplementary material for: Flexynesis: A deep learning toolkit for bulk multi-omics data integration for precision oncology and beyond
Source: Nat Commun. 2025 Sep 12;16:8261. doi: 10.1038/s41467-025-63688-5 (PMC12432156; doi:10.1038/s41467-025-63688-5)

Supplementary Figure 1:

A schematic representation of how multi-modal tabular data input is processed and prepared for training models in Flexynesis. See “Methods/Importing the training and test datasets” for a detailed description of the data processing steps.

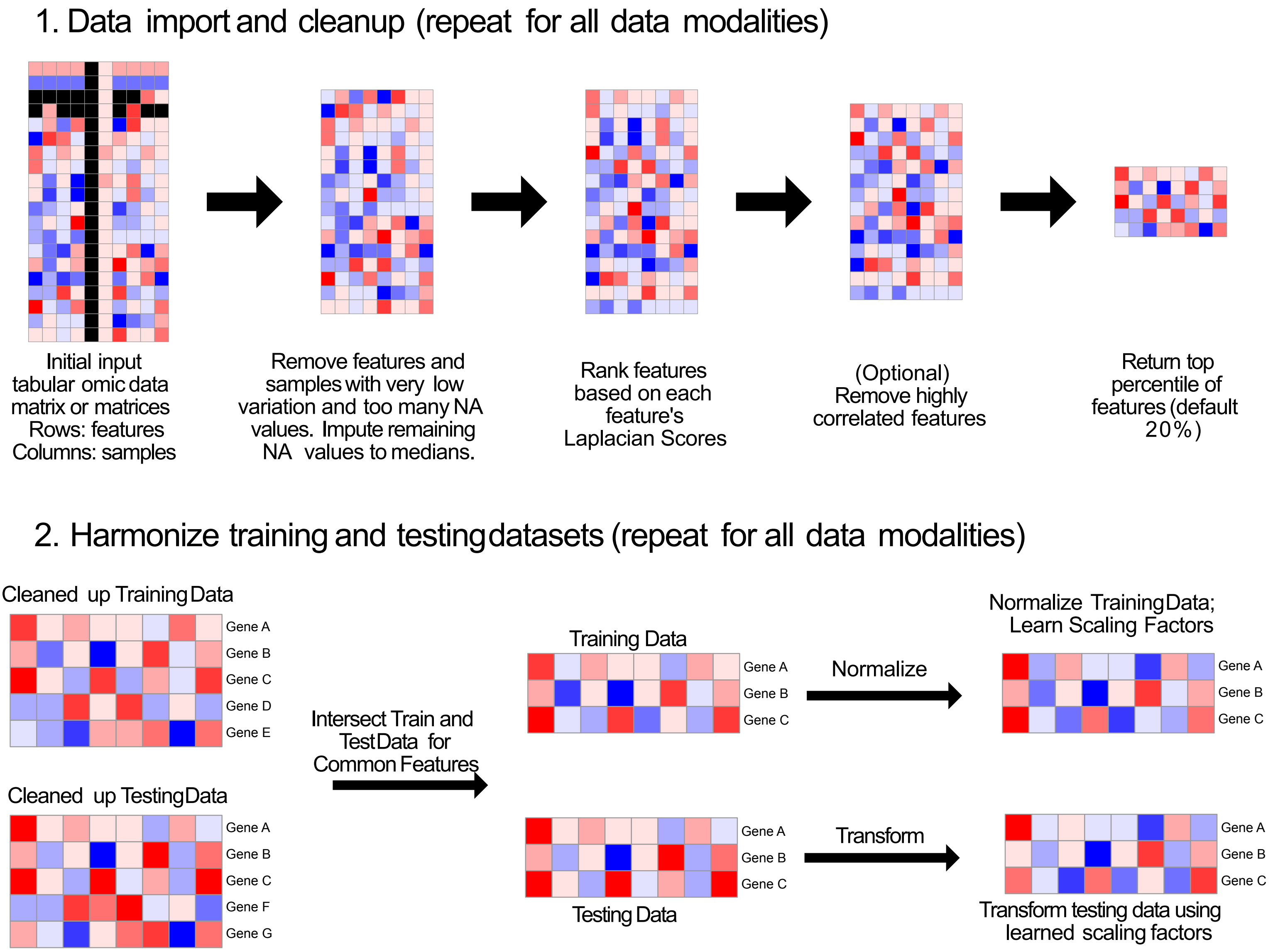

Supplementary Figure 2:

A schematic representation of the sequential Bayesian hyperparameter optimization routine used in training models in Flexynesis. For more details, see the "Methods/Hyperparameter optimization" section.

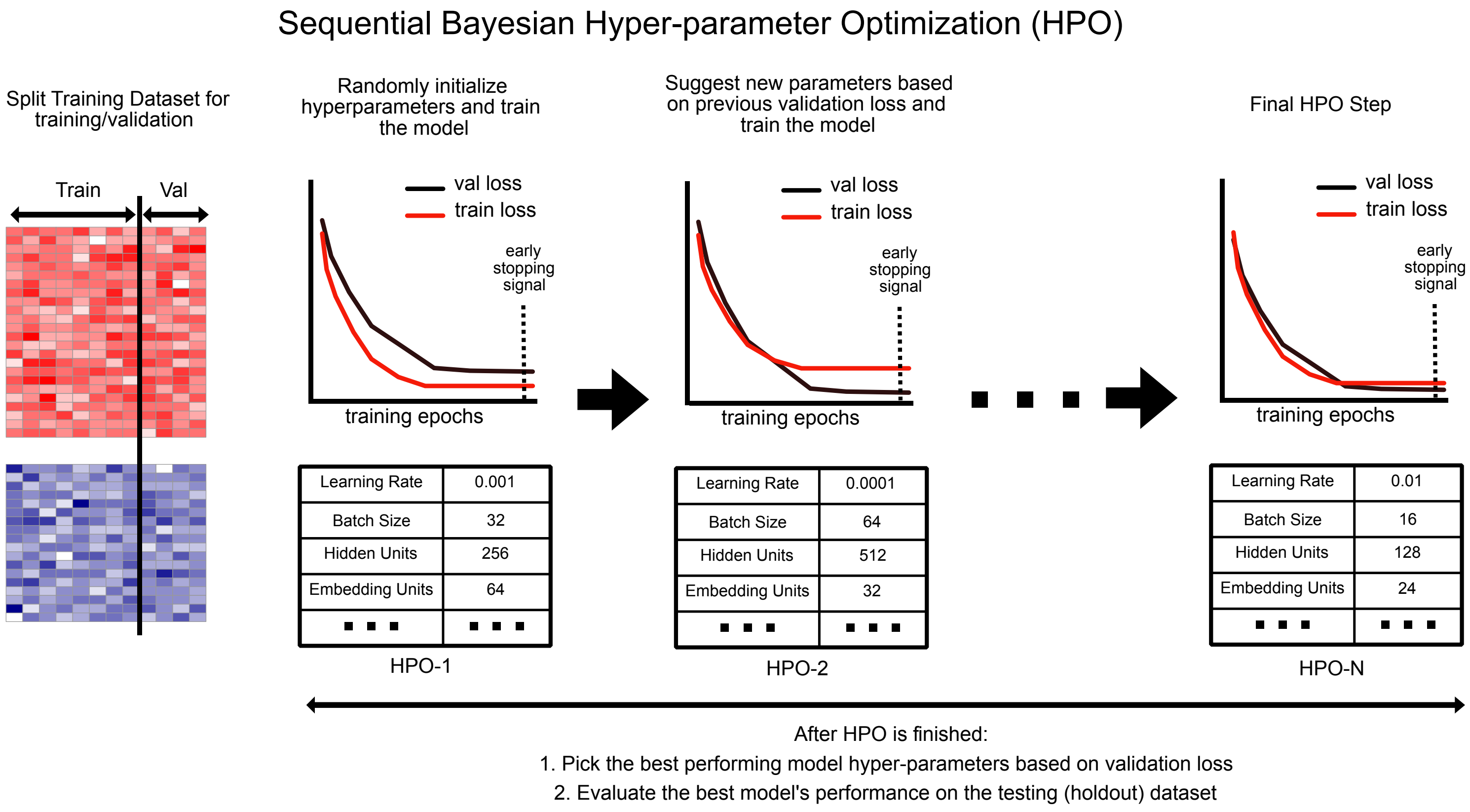

Supplementary Figure 3:

A schematic representation of how a pre-trained model can be fine-tuned in Flexynesis. For more details, see the “Methods/Model fine-tuning” section.

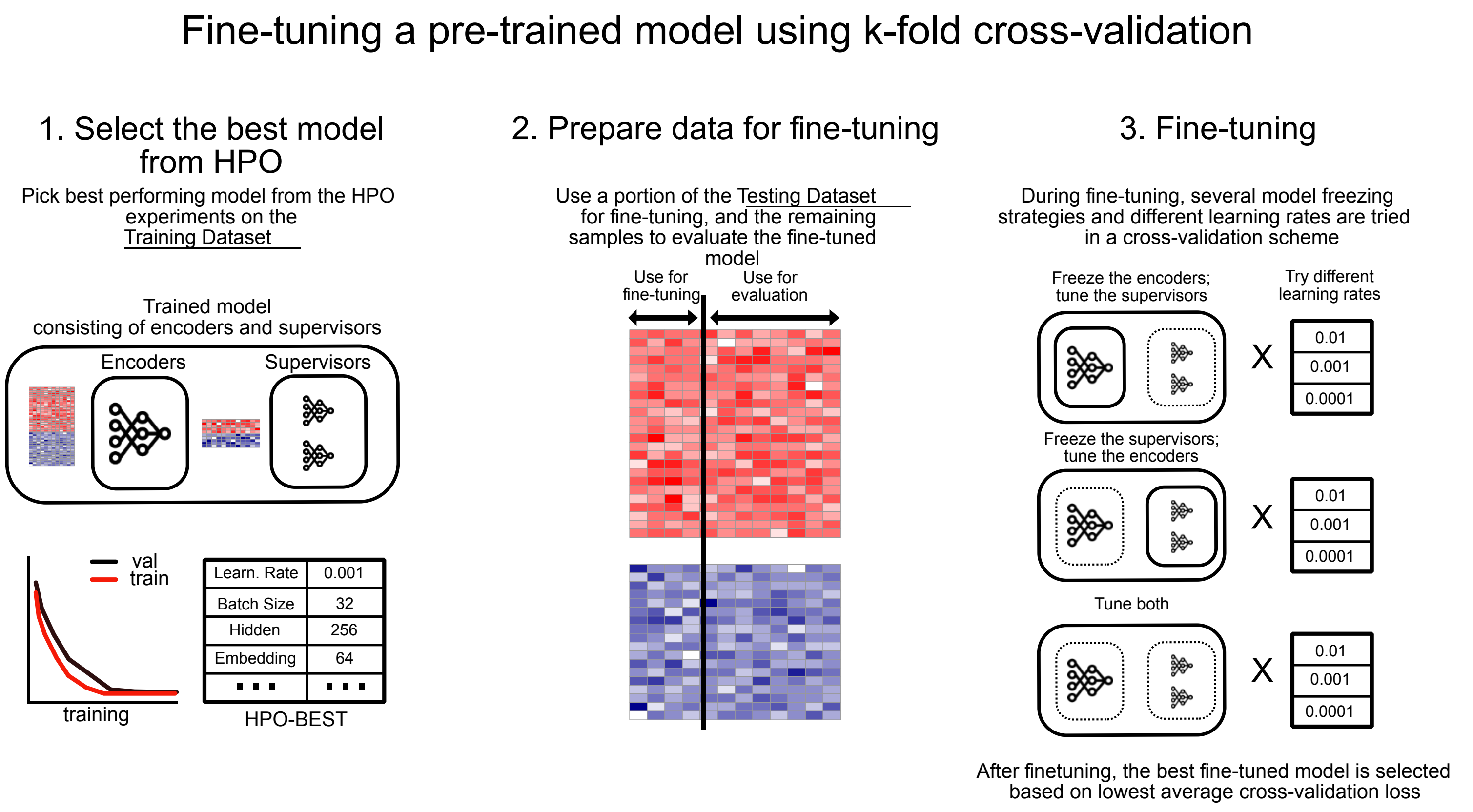

Supplementary Figure 4:

A schematic representation of the DirectPred architecture in Flexynesis. Multi-modal data input (optionally including encoded covariate matrices) is first processed using dedicated multi-layer perceptrons (MLPs) to create a latent feature representation of the samples (sample embeddings). These embeddings are concatenated and used as input to a dedicated MLP, depending on the target variables of interest. See the “Methods/Model training and loss functions” section for a detailed description of the different types of loss functions used according to the target variable types. Additionally, the sample embeddings obtained post-training can be used for clustering the samples.

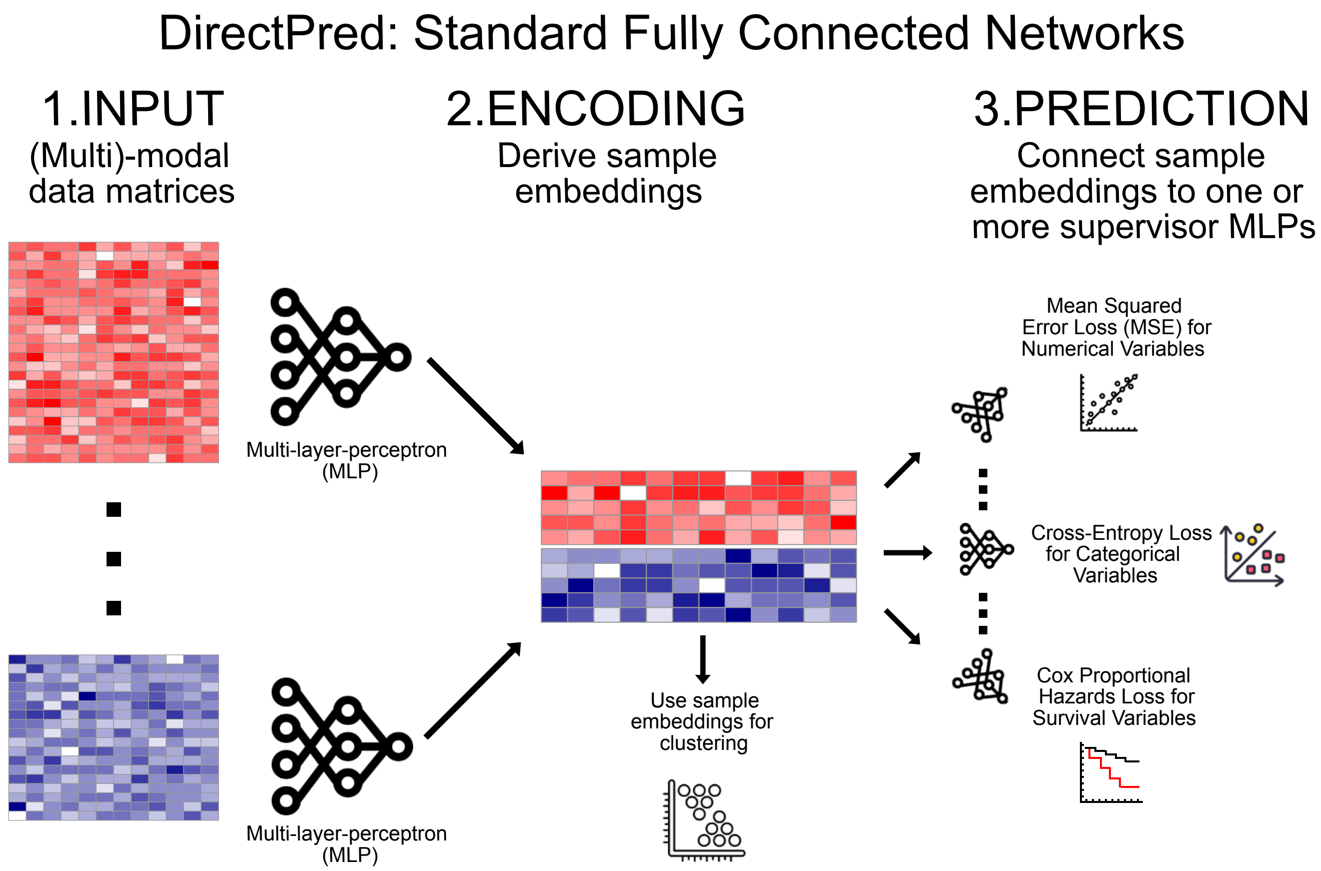

Supplementary Figure 5:

A schematic representation of the supervised_vae (supervised variational auto-encoder) architecture in Flexynesis. In this model, multi-modal data matrices are encoded to obtain sample embeddings, which are then decoded to reconstruct the input data matrices. An MMD loss is calculated as a regularization term for the sample embedding space, while a negative log-likelihood loss is computed to represent the distance between the reconstructed/decoded output and the multi-modal input. The sample embeddings can later be connected to dedicated MLPs for each target variable of interest. See the "Methods/Model training and loss functions" section for a detailed description of the different loss functions used according to target variable types. Furthermore, the sample embeddings obtained post-training can also be used for clustering the samples.

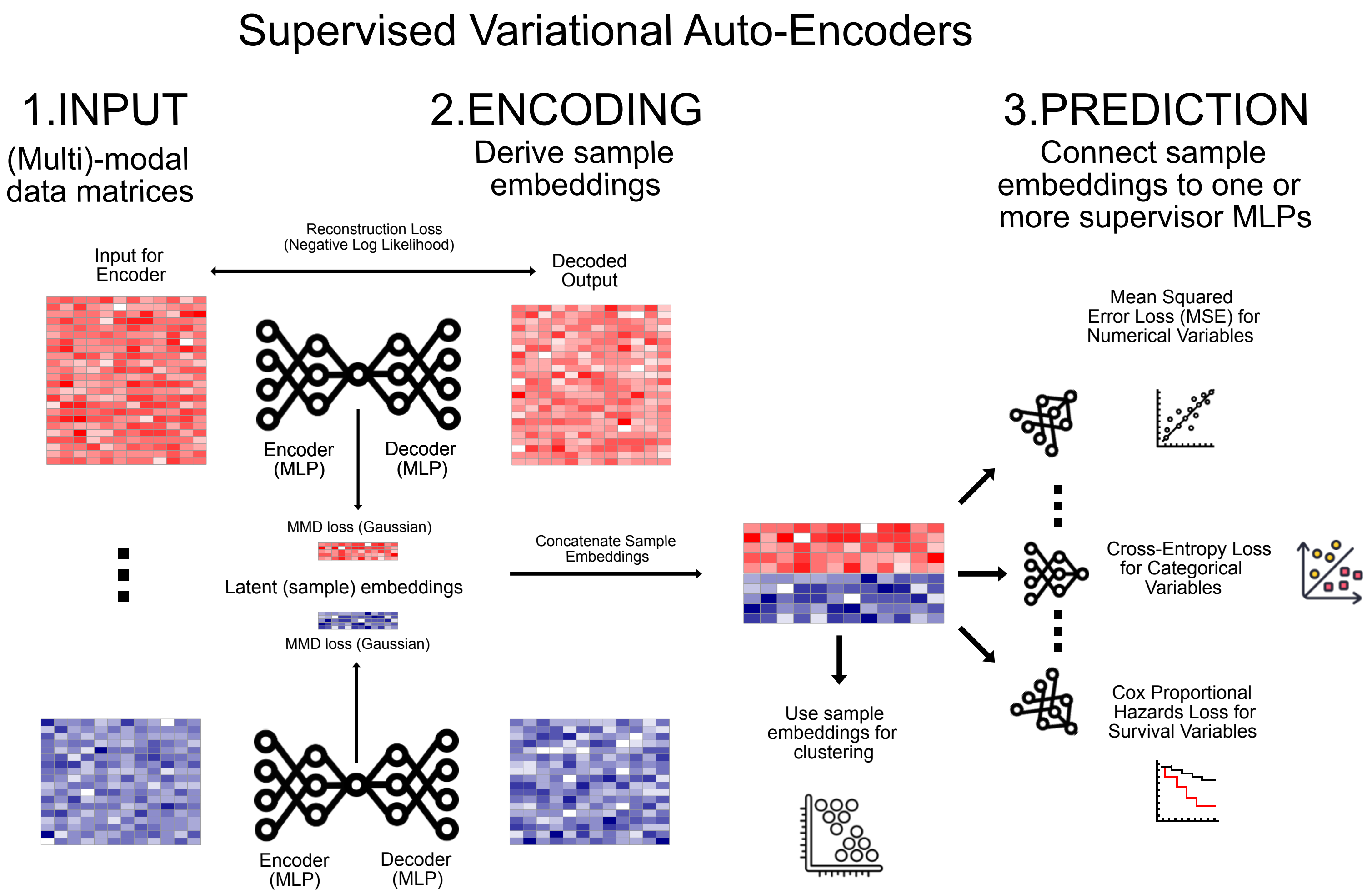

Supplementary Figure 6:

A schematic representation of the CrossModalPred (cross-modality encoder/prediction networks) architecture in Flexynesis. This model can be used to translate one or more data matrices (blue and red) into a target data matrix (green) of interest. Multi-modal data matrices are encoded to obtain sample embeddings, which are then decoded to reconstruct a target data matrix. An MMD loss is applied as a regularization term for the sample embedding space, and a negative log-likelihood loss is computed to measure the distance between the reconstructed/decoded output and the target matrix. The sample embeddings can later be connected to dedicated MLPs for each target variable of interest. See the "Methods/Model training and loss functions" section for a detailed description of the different types of loss functions used according to target variable types. Additionally, the sample embeddings obtained post-training can be used for clustering the samples.

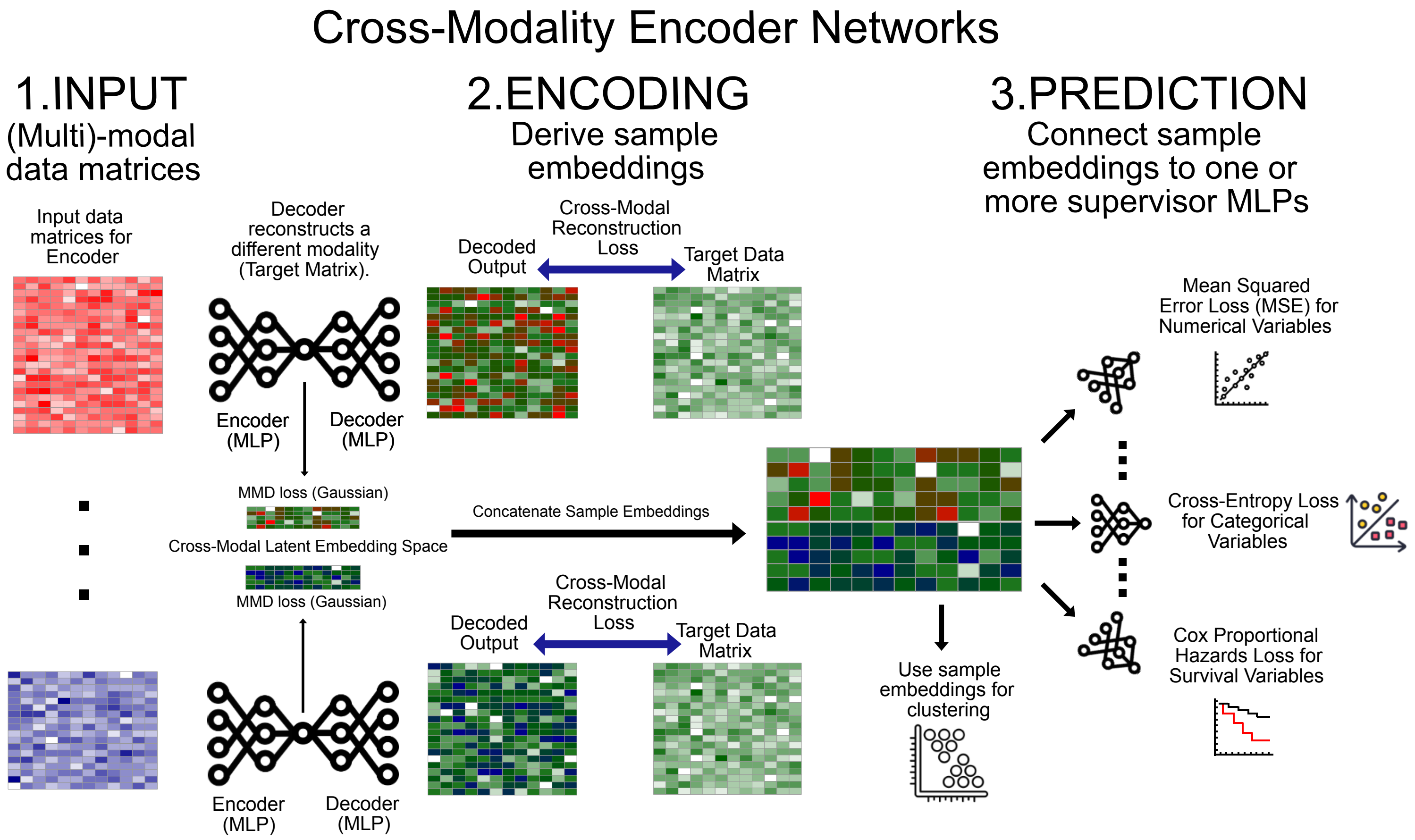

Supplementary Figure 7:

A schematic representation of the Multi-Triplet-Networks architecture in Flexynesis. In this model, for each sample, a triplet of samples is constructed: the current sample serves as the “anchor”, a “positive” sample is randomly drawn from the same class as the anchor, and a “negative” sample is randomly drawn from a different class. For each triplet (anchor, positive, negative), a dedicated MLP generates sample embeddings. A triplet loss is calculated to maximize the distance between the anchor and negative samples while minimizing the distance between the anchor and positive samples. The sample embeddings for the anchor samples are concatenated and connected to dedicated MLPs for each target variable of interest. See the “Methods/Model training and loss functions” section for a detailed description of the different loss functions used according to target variable types. Furthermore, the sample embeddings obtained post-training can be used for clustering the samples.

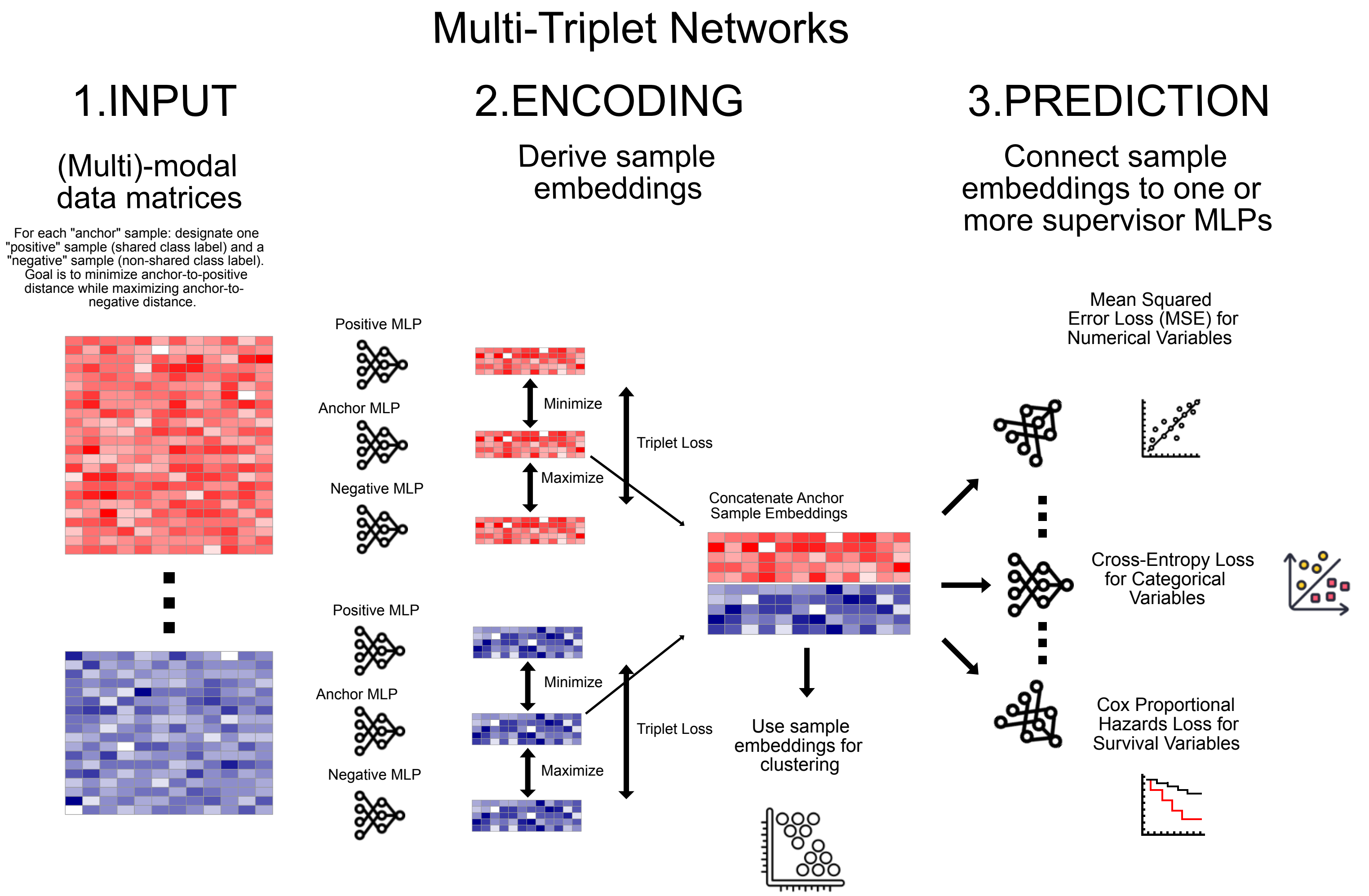

Supplementary Figure 8:

A schematic representation of the GNN (Graph Neural Networks) architecture in Flexynesis. Multi-modal input data matrices are overlaid on the STRING database of gene-gene interactions, where each gene is represented as a node in the network. The node features correspond to the feature values from the omic data modalities. These node features are transformed into node embeddings using one or more graph convolutional layers (GCN/GC/SAGE options available). The resulting node embeddings are concatenated and further processed with an MLP to obtain sample embeddings. The sample embeddings for the anchor samples are concatenated and connected to dedicated MLPs for each target variable of interest. See the "Methods/Model training and loss functions" section for a detailed description of the different types of loss functions used according to target variable types. Additionally, the sample embeddings obtained post-training can be used for clustering the samples.

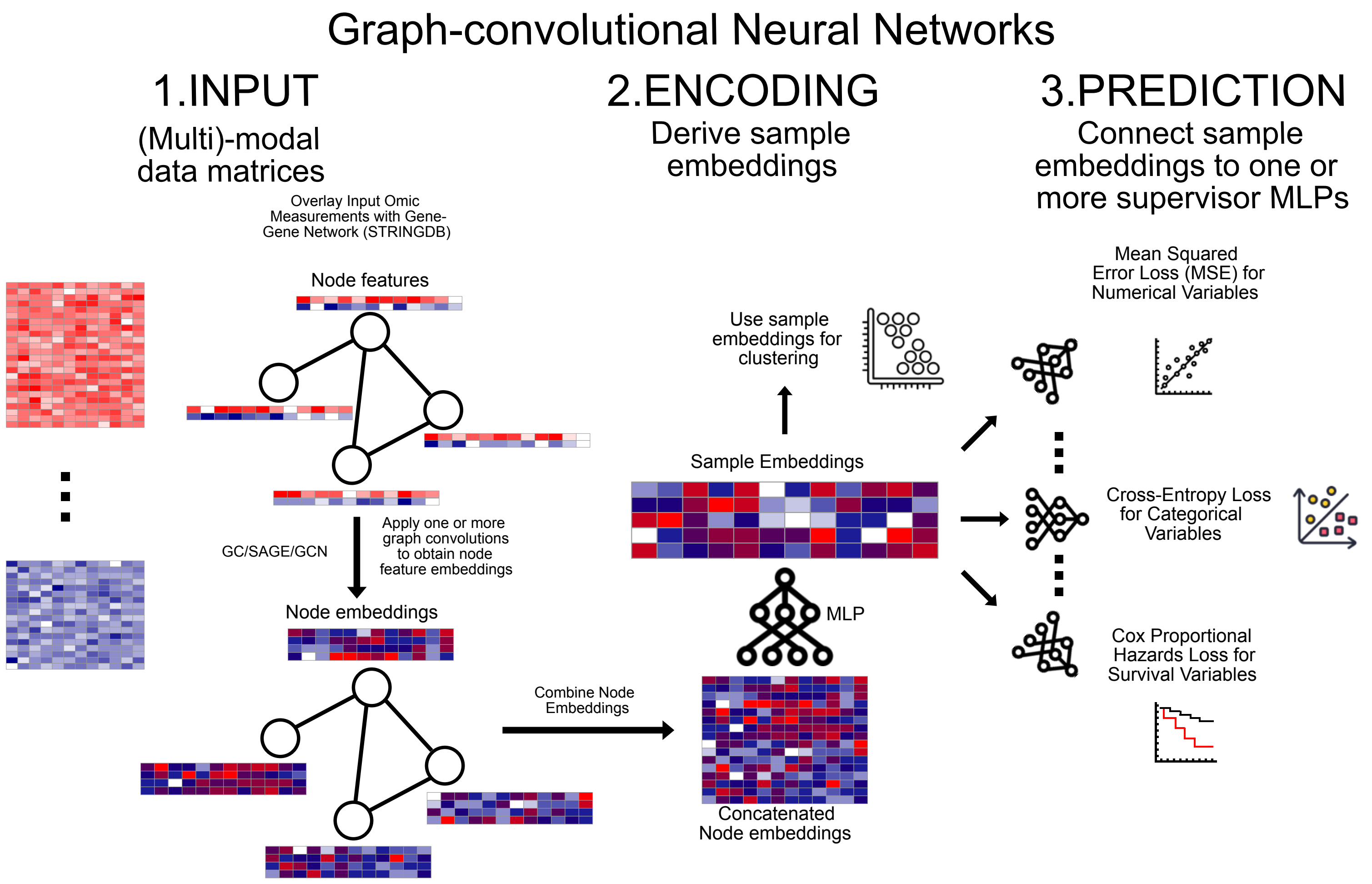

Supplementary Figure 9:

Comparison of feature attribution scores obtained from the two methods: Integrated Gradients and GradientSHAP for the drug response biomarker discovery analysis (see Figure 7B).

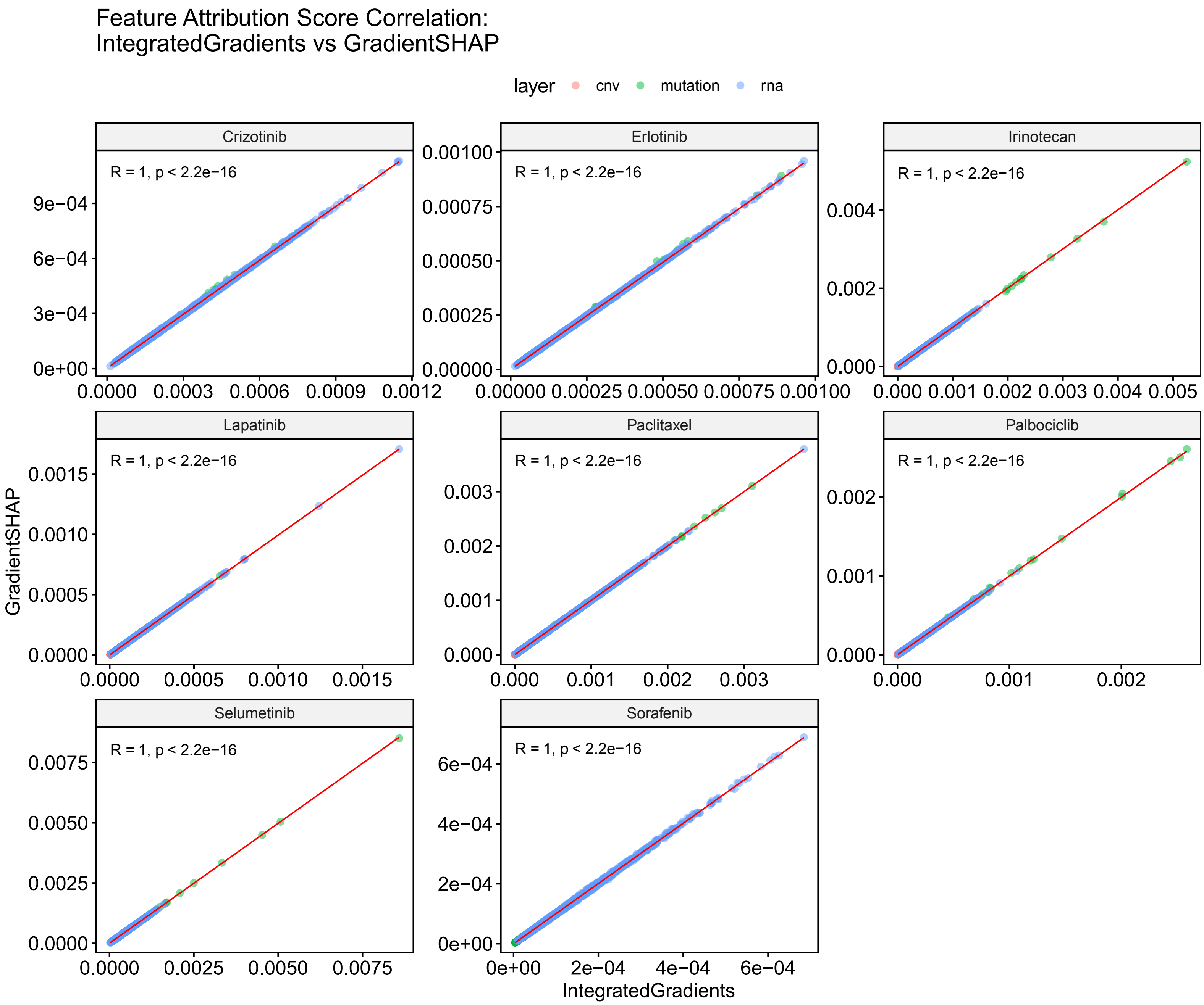

Supplementary Figure 10:

Runtime (wall clock times) (panel A) and peak RAM consumption in CPU (panel B) and GPU (panel C) of different deep learning architectures using a typical bulk multi-omics dataset (500 breast cancer samples with two data modalities with 2000 features each) for data processing and a single hyperparameter set with different multi-modal data fusion options (intermediate / early).

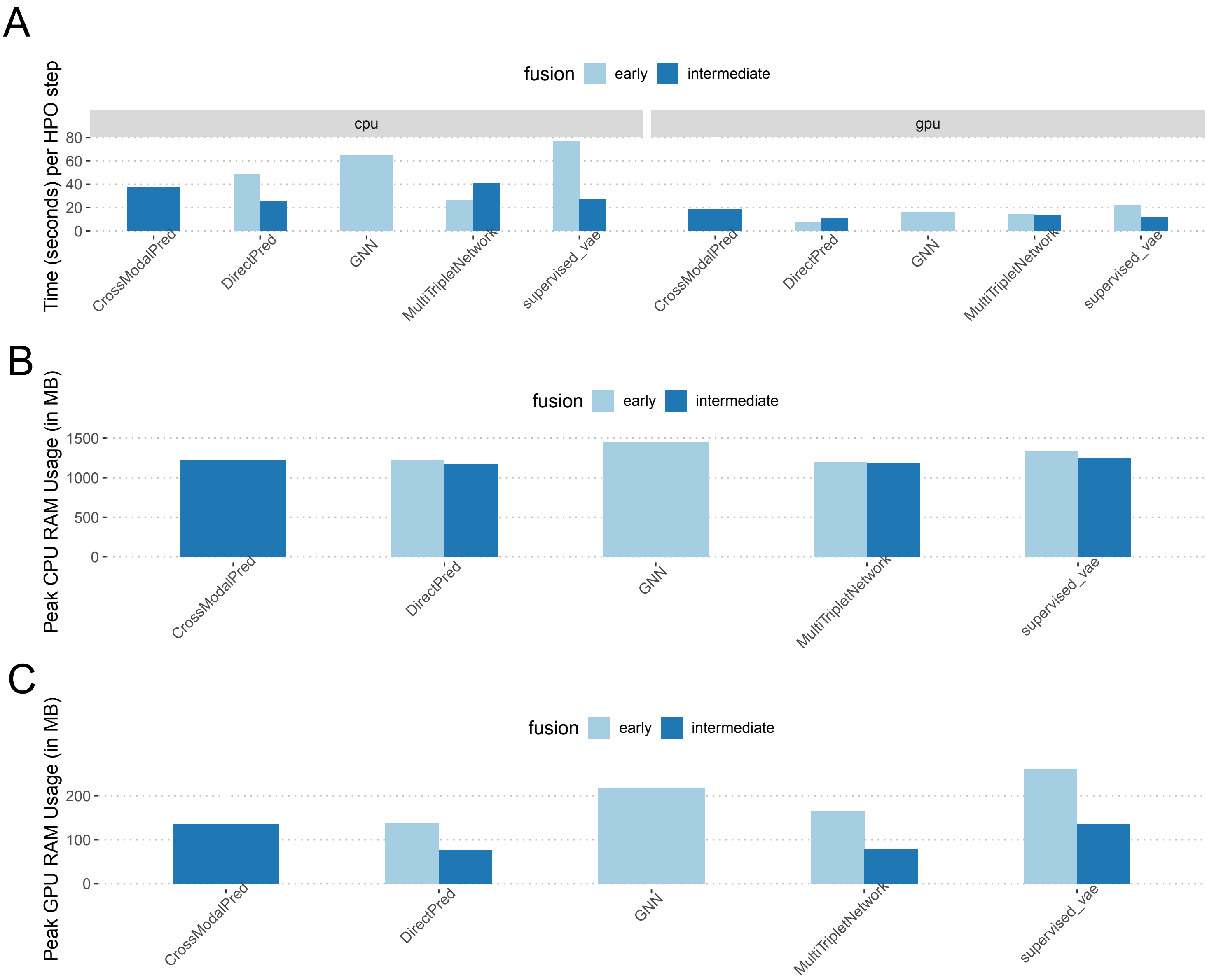

Supplement: Supplementary file 1 — Supplementary Information [file 41467_2025_63688_MOESM1_ESM.pdf]
