# Supplementary material for: Flexynesis: A deep learning toolkit for bulk multi-omics data integration for precision oncology and beyond
Source: Nat Commun. 2025 Sep 12;16:8261. doi: 10.1038/s41467-025-63688-5 (PMC12432156; doi:10.1038/s41467-025-63688-5)
Supplement: Supplementary file 3 — Description of Additional Supplementary Files [file 41467_2025_63688_MOESM3_ESM.pdf]

Description of Additional Supplementary Files

Supplementary Data 1:
A literature survey of published multi-omics data integration methods.

Supplementary Data 2:
Benchmarking statistics for MSI status prediction in gastrointestinal and gynecological cancers using different deep learning architectures and fusion options.

Supplementary Data 3:
Source data with respect to Figure 5C. The raw matrix of the number of samples with respect to cluster memberships and known cancer type labels.

Supplementary Data 4:
Source data with respect to Figure 6. Paired bootstrap test statistics comparing the best performing fine-tuned deep learning architecture against non-fine-tuned deep learning architectures and baseline methods.

Supplementary Data 5:
Source data with respect to Figure 7. Sheet1: model performance statistics for the best performing models (Figure 7A). Sheet2: feature attribution scores for the top 10 markers detected for each drug (Figure 7B).

Supplementary Data 6:
Source data with respect to Figure 8. Sheet1: model performance metrics from the benchmarking experiments across 14 different multi-omics data integration tasks. Sheet2: paired two-sided bootstrap t-test statistics comparing the best performing baseline methods against the best performing deep learning architectures in each of the 14 tasks.

Supplementary Data 7:
Source data with respect to Supplementary Figure 10. Runtime (wall clock times) (panel A) and peak RAM consumption in CPU (panel B) and GPU (panel C) of different deep learning architectures using a typical bulk multi-omics dataset (500 breast cancer samples with two data modalities with 2000 features each) for data processing and a single hyperparameter set with different multi-modal data fusion options (intermediate / early).
