# Supplementary material for: Flexynesis: A deep learning toolkit for bulk multi-omics data integration for precision oncology and beyond
Source: Nat Commun. 2025 Sep 12;16:8261. doi: 10.1038/s41467-025-63688-5 (PMC12432156; doi:10.1038/s41467-025-63688-5)
Supplement: Supplementary file 11 — Reporting Summary [file 41467_2025_63688_MOESM11_ESM.pdf]

nature portfolio

Corresponding author(s): Bora Uyar, Altuna Akalin

Last updated by author(s): 25.08.2025

Reporting Summary

Nature Portfolio wishes to improve the reproducibility of the work that we publish. This form provides structure for consistency and transparency in reporting. For further information on Nature Portfolio policies, see our Editorial Policies and the Editorial Policy Checklist.

Statistics

For all statistical analyses, confirm that the following items are present in the figure legend, table legend, main text, or Methods section.

n/a	Confirmed	
☐	☒	The exact sample size (*n*) for each experimental group/condition, given as a discrete number and unit of measurement
☐	☒	A statement on whether measurements were taken from distinct samples or whether the same sample was measured repeatedly
☐	☒	The statistical test(s) used AND whether they are one- or two-sided<br>*Only common tests should be described solely by name; describe more complex techniques in the Methods section.*
☐	☒	A description of all covariates tested
☒	☐	A description of any assumptions or corrections, such as tests of normality and adjustment for multiple comparisons
☐	☒	A full description of the statistical parameters including central tendency (e.g. means) or other basic estimates (e.g. regression coefficient) AND variation (e.g. standard deviation) or associated estimates of uncertainty (e.g. confidence intervals)
☐	☒	For null hypothesis testing, the test statistic (e.g. *F*, *t*, *r*) with confidence intervals, effect sizes, degrees of freedom and *P* value noted<br>*Give P values as exact values whenever suitable.*
☒	☐	For Bayesian analysis, information on the choice of priors and Markov chain Monte Carlo settings
☒	☐	For hierarchical and complex designs, identification of the appropriate level for tests and full reporting of outcomes
☐	☒	Estimates of effect sizes (e.g. Cohen's *d*, Pearson's *r*), indicating how they were calculated

Our web collection on statistics for biologists contains articles on many of the points above.

Software and code

Policy information about availability of computer code

Data collection	The description of datasets, how to access and process the data: https://zenodo.org/records/16444303
Data analysis	The source code repositories developed for this study at the time of this manuscript submission are deposited at the Zenodo database:<br><br>The raw/processed dataset descriptions along with code to reproduce manuscript figures can be found at https://github.com/BIMSBbioinfo/flexynesis_manuscript. (v.1.0.3 is available at https://zenodo.org/records/16444303). The repo is accessible with an MIT licence.<br><br>Core Flexynesis software package is available at: https://github.com/BIMSBbioinfo/flexynesis (v1.0.0 available at https://zenodo.org/records/16444460). The repo is accessible for free for academic usage, but restricted for commercial purposes.<br><br>The accessory benchmarking pipeline utilizing Flexynesis is available at: https://github.com/BIMSBbioinfo/flexynesis-benchmarks (v.1.0.0 is available at https://zenodo.org/records/16443659) . The repo is accessible for free for academic usage, but restricted for commercial purposes.

For manuscripts utilizing custom algorithms or software that are central to the research but not yet described in published literature, software must be made available to editors and reviewers. We strongly encourage code deposition in a community repository (e.g. GitHub). See the Nature Portfolio guidelines for submitting code & software for further information.

Data

Policy information about availability of data

All manuscripts must include a data availability statement. This statement should provide the following information, where applicable:

- Accession codes, unique identifiers, or web links for publicly available datasets
- A description of any restrictions on data availability
- For clinical datasets or third party data, please ensure that the statement adheres to our policy

All the datasets used in this study are previously published datasets (see Methods for how they were further processed for training). None of these datasets are under restricted accesss:
Multi-omic and drug response data for the cell lines from the CCLE 10 can be downloaded from https://zenodo.org/records/3905462.
Multi-omic and drug response data for the cell lines from the GDSC can be downloaded from https://zenodo.org/record/3905481.
The merged cohorts for Lower Grade Glioma (LGG) and Glioblastoma MultiForme (GBM) dataset 21 are available at: https://www.cbioportal.org/study/summary?id=lgggbm_tcga_pub.
Multi-omics data for colorectal cancer (TCGA) are available at Cbioportal: https://www.cbioportal.org/study/summary?id=coadread_tcga_pan_can_atlas_2018.
Multi-omics data for breast invasive carcinoma (TCGA) are available at Cbioportal: https://www.cbioportal.org/study/summary?id=brca_tcga_pan_can_atlas_2018.
Multi-omics data for glioblastoma multiforme 21 are available at Cbioportal: https://www.cbioportal.org/study/summary?id=gbm_tcga_pan_can_atlas_2018.
Multi-omic data for the metastatic breast cancer cohort from the METABRIC study 23 are available at Cbioportal: https://www.cbioportal.org/study/summary?id=brca_metabric
Single-cell CITE-Seq dataset 63 is available via the Seurat (v5.1.0) 64 package.
The omics data, CRISPR screens and PRISM drug screening data for cell lines from the DepMap project 26 is available at the DepMap Portal (https://DepMap.org/portal).
The TCGA datasets are available via the TCGABiolinks package 65.
Structural/functional features of human protein sequences are available at the describePROT database 30: http://biomine.cs.vcu.edu/servers/DESCRIBEPROT/download_database_value/9606_value.csv.
All the above-mentioned datasets were further processed to enable training Flexynesis models. The datasets prepared for training Flexynesis models and the outputs of the model training required to reproduce the figures and tables in this manuscript are available in the Zenodo database under DOI accession 10.5281/zenodo.16442997 (https://zenodo.org/records/16442998). The source data for the figures are provided with this paper.

Research involving human participants, their data, or biological material

Policy information about studies with human participants or human data. See also policy information about sex, gender (identity/presentation), and sexual orientation and race, ethnicity and racism.

Reporting on sex and gender	Not applicable. Only publicly available datasets were utilized in this study.
Reporting on race, ethnicity, or other socially relevant groupings	Not applicable. Only publicly available datasets were utilized in this study.
Population characteristics	Not applicable. Only publicly available datasets were utilized in this study.
Recruitment	Not applicable. Only publicly available datasets were utilized in this study.
Ethics oversight	Not applicable. Only publicly available datasets were utilized in this study.

Note that full information on the approval of the study protocol must also be provided in the manuscript.

Field-specific reporting

Please select the one below that is the best fit for your research. If you are not sure, read the appropriate sections before making your selection.

☒ Life sciences ☐ Behavioural & social sciences ☐ Ecological, evolutionary & environmental sciences

For a reference copy of the document with all sections, see nature.com/documents/nr-reporting-summary-flat.pdf

Life sciences study design

All studies must disclose on these points even when the disclosure is negative.

Sample size	Sample sizes for various datasets utilized in this study are described in the methods section.
Data exclusions	Data processing steps and exclusion criteria for samples are described in the methods section and also available under https://github.com/BIMSBbioinfo/flexynesis
Replication	All data processing and analysis steps along with how to reproduce the figures in the manuscript are detailed in the methods section and under the github repository: https://github.com/BIMSBbioinfo/flexynesis_manuscript

Randomization	Samples were randomly assigned to train/test/validation splits. Details are documented in the methods section and under the github repository: https://github.com/BIMSBbioinfo/flexynesis_manuscript
Blinding	Blinding was not possible, because we only did retrospective data analysis on publicly available data. However, we followed machine learning best practices to avoid data leakage by enforcing train/validation/test splits and evaluation of methods on held-out data.

Reporting for specific materials, systems and methods

We require information from authors about some types of materials, experimental systems and methods used in many studies. Here, indicate whether each material, system or method listed is relevant to your study. If you are not sure if a list item applies to your research, read the appropriate section before selecting a response.

Materials & experimental systems

n/a	Involved in the study
☒	☐ Antibodies
☒	☐ Eukaryotic cell lines
☒	☐ Palaeontology and archaeology
☒	☐ Animals and other organisms
☒	☐ Clinical data
☒	☐ Dual use research of concern
☒	☐ Plants

Methods

n/a	Involved in the study
☒	☐ ChIP-seq
☒	☐ Flow cytometry
☒	☐ MRI-based neuroimaging

Plants

Seed stocks	*Report on the source of all seed stocks or other plant material used. If applicable, state the seed stock centre and catalogue number. If plant specimens were collected from the field, describe the collection location, date and sampling procedures.*
Novel plant genotypes	*Describe the methods by which all novel plant genotypes were produced. This includes those generated by transgenic approaches, gene editing, chemical/radiation-based mutagenesis and hybridization. For transgenic lines, describe the transformation method, the number of independent lines analyzed and the generation upon which experiments were performed. For gene-edited lines, describe the editor used, the endogenous sequence targeted for editing, the targeting guide RNA sequence (if applicable) and how the editor was applied.*
Authentication	*Describe any authentication procedures for each seed stock used or novel genotype generated. Describe any experiments used to assess the effect of a mutation and, where applicable, how potential secondary effects (e.g. second site T-DNA insertions, mosiacism, off-target gene editing) were examined.*
